# Empowering Retailers to Refuse to Sell Tobacco Products to Minors

**DOI:** 10.3390/ijerph15020245

**Published:** 2018-02-01

**Authors:** Min-Li Chen, Li-Na Chou, Ya-Cheng Zheng

**Affiliations:** 1 Department of Respiratory Care and Graduate Institute of Nursing, Chang Gung University of Science and Technology, Chiayi 61363, Taiwan; t73003@gmail.com; 2 Department of Nursing, National Tainan Junior College of Nursing, Tainan 700, Taiwan; 3 Graduate Institute of Nursing, Chang Gung University of Science and Technology, Chiayi 61363, Taiwan; s2006888@cgmh.org.tw; 4 Chiayi Chang Gung Memorial Hospital, Chiayi 61363, Taiwan

**Keywords:** empowerment, tobacco product retailer, adolescent

## Abstract

Tobacco smoking and exposure to secondhand smoke are major environmental risk factors that negatively influence health. It is recommended that tobacco product manufacturers and retailers receive empowerment counseling programs to achieve adolescent health targets. The present study conducted an empowerment counseling session to counsel tobacco product retailers in refusing to sell tobacco products to minors. The three stages of this study were conducted from March 2015 to February 2017. The results revealed that 74% of retailers were selling tobacco products to minors at baseline, 40% at stage two and 15% at stage three. These represent significant reductions in selling tobacco products to minors (all stage differences *p* < 0.001). However, experimental design studies could be used to examine the empowerment counseling program for preventing tobacco sales to minors in the future. Moreover, health care providers should collaborate with tobacco product retailers to design unique empowerment counseling sessions for specific regions to improve retailers’ capabilities for self-management in terms of tobacco hazard prevention.

## 1. Introduction

Cigarette smoking is a growing problem among Taiwanese adolescents. According to the 2016 health statistics released by the Ministry of Health and Welfare, Taiwan, the cigarette smoking population among people less than 18 years old comprised 3.7% of all junior high students and 9.3% of all senior high school students [1]. Tobacco smoking is a major source of air pollution and ambient particulate matter 2.5 (PM2.5) [2,3]. Tobacco smoking and exposure to secondhand smoke are major environmental risk factors that negatively influence adolescent health. Many studies have shown that air pollution caused by teenagers smoking can damage the health of not only adolescents who smoke but also those who do not smoke through exposure to secondhand smoke [4,5]. In an effort to mitigate the potential impact of tobacco on adolescent health, the WHO has announced regulations to forbid the selling of tobacco products to minors [6]. In 1997, Taiwan’s government announced Article 13 of the Tobacco Hazards Prevention Act, which stipulates that no person shall provide tobacco products to people under the age of 18. However, although tobacco product sales to people aged less than 18 years are forbidden in Taiwan, many adolescents still manage to purchase tobacco products easily. In 2015, a survey on tobacco product accessibility and availability for adolescents was conducted by the Health Promotion Administration, Ministry of Health and Welfare, Taiwan. The results showed that 52.9% of junior high school student smokers were not refused by retailers when attempting to purchase tobacco products, and the most frequently visited locations for tobacco product purchases were traditional grocery stores (58.3%) [7]. Moreover, the results showed that among the senior high school student smokers, 67.1% were not refused tobacco products by retailers, and convenience stores were the most frequently visited locations for purchases (51.4%) [7]. The same survey showed that in 48.5% of tobacco product sales, the retailers did not check customer ages and directly sold tobacco products to adolescents [7]. In 2017, 234 tobacco product retailers in 18 towns in Southern Taiwan were surveyed by the Department of Health Bureau through random sampling. The survey findings revealed that traditional grocery stores (51.7%) and betel nut stalls (24.60%) were the most common types of retailers to sell tobacco products to adolescents aged less than 18 years without checking their age [8]. Adolescent tobacco-related hazard prevention should focus on not only promoting related knowledge among adolescents, but also encouraging self-management among tobacco product retailers to reduce the sales of tobacco products to people aged less than 18 and improve adolescent health and air quality. These tasks are urgent objectives for public health care providers.

Studies have shown that counseling programs can change retailer knowledge, attitudes, and behaviors related to cigarette sales [9] and restrict tobacco sales to youths [10]. Applying the empowerment concept to assist people in developing the critical capability of self-awareness, recognizing their personal situations, and improving their problem-solving capabilities enables them to have more control over their daily lives [11]. The empowerment process involves building partnerships, listening, dialogue, reflection, action, and feedback [11]. Empowerment is based on the concept of equality and mutual respect between facilitators and participants, and is used to initiate interactive discussions where participants can be assisted in identifying and clarifying personal problems and solutions by using their own powers and capabilities [12]. Empowerment is applied in health care to improve adolescents’ level of self-control in terms of health, and prevent adverse health outcomes caused by tobacco consumption. Studies have indicated that empowerment strategies should focus on identifying and building adolescents’ capacity and strength to reduce their tobacco use, thereby facilitating their avoidance of harmful or risky behavior [13,14]. Scholars have suggested that applied empowerment on tobacco product manufacturers and retailers reduce tobacco sales to consumers [15]. To protect adolescent health and maintain a healthy environment, empowerment applied by health care providers to increase self-awareness among tobacco product retailers who sell to people aged less than 18 years should be the focus in order to be effective. However, few studies have examined the effectiveness of empowerment counseling programs among tobacco retailers that refuse to sell tobacco products to minors. The present study included an empowerment counseling session to counsel tobacco product retailers in Southern Taiwan in refusing to sell tobacco products to people aged less than 18 years.

## 2. Materials and Methods

As part of this study, researchers were authorized by the Department of Health Bureau in Southern Taiwan to conduct an empowerment counseling session to counsel tobacco product retailers in refusing to sell tobacco products to people aged less than 18 years. Based on the study objective, the Institutional Review Board (IRB) review process could be waived because of legal orders (Exempt Review of IRB No. 201700685B1).

### 2.1. Designs and Sampling

As part of this study, an empowerment counseling session was conducted to counsel tobacco product retailers in refusing to sell tobacco products to adolescents aged less than 18 years. This study was conducted from March 2015 to February 2017, and involved an investigation of retailers selling tobacco products to people aged less than 18 years in 18 towns in Southern Taiwan. A one-group pretest-posttest design was conducted. The number of samples of tobacco retailers was based on the population of the county in this study; 25 and 15 tobacco retailers were selected in towns with populations of more and less than 50,000, respectively. Thus, 327 tobacco retailers were selected in accordance with the percentages of grocery stores, betel nut stalls, and convenience stores. In total, 327 tobacco products retailers including grocery stores, betel nut stalls, and convenience stores, located in mountainous, coastal, and plain regions in Southern Taiwan, were recruited in this study. The study testers were university students aged over 18 years who wore uniforms to disguise themselves as high school students. To ensure the consistency in observation, every study tester underwent a two-hour meeting regarding the study purpose and design. They were assigned the task of purchasing tobacco products from 327 tobacco retailers in Southern Taiwan and assessed whether the retailers requested proof of age before selling tobacco products.

This study was conducted in three stages. The first stage, conducted in March 2015, assessed whether the aforementioned 327 retailers sold tobacco products to people aged less than 18 years. Subsequently, related government authorities issued documents to retailers and instructed them to receive the empowerment counseling session (Table 1). The second stage of this study was conducted in March 2016 following six months of empowerment counseling sessions. In this stage, we continued to conduct investigations on the 327 retailers. Retailers that sold tobacco products to minors in the first stage and did so again in the second stage were to receive empowerment counseling sessions. After a second six months of empowerment counseling sessions, we conducted the third stage in November 2016. During this stage, we investigated all 327 retailers to determine whether they sold tobacco products to minors. Counseling was provided by registered nurses of the health bureau, counseling volunteers consisted of retired registered nurses, and people who had completed training courses for tobacco hazard prevention.

The empowerment counseling session employed in this study was as follows: (1) building partnerships between tobacco product retailers and health care providers to promote mutual respect, trust, sharing, equality and joint participation and enhance their motivation for self-awareness; (2) listening to tobacco product retailers to understand what they care about, demand, and expect; (3) initiating dialogue and discussions involving tobacco product retailers to clarify their concerns and the resources they require and enhance their understanding of the importance of not selling tobacco products to people aged less than 18 years; (4) encouraging tobacco product retailers to develop skills of critical thinking and good decision-making through reflection and reviewing their self-management experiences; (5) assisting tobacco product retailers in establishing an action plan to thoroughly execute self-management and make behavioral changes to achieve the goal of “No selling tobacco products to people aged less than 18 years”; and (6) continuing to provide feedback, share, and maintain motivation and behavior in not selling tobacco products to people aged less than 18 years (Table 1).

### 2.2. Statistical Analysis

SPSS Statistics 20.0 (SPSS Inc., Chicago, IL, USA) was used for the statistical analysis. Descriptive statistics (frequency distributions and percentages) were used to determine which tobacco retailers sold tobacco products to minors. The Chi-squared test and McNemar’s test were conducted to test for differences between the retailers that sold tobacco products to minors and those who did not.

## 3. Results

The retailers were divided into eight groups based on whether they sold or refused to sell tobacco products to minors (Table 2) at stage one (baseline), at stage two (after retailers selling tobacco products to minors had experienced one intervention) and at stage three (after retailers selling tobacco products to minors at stage one had experienced the stage two intervention). The largest group (41%) stopped selling tobacco products to minors after one intervention. The second largest group (25%) stopped selling tobacco products to minors after two interventions. Thus, two thirds of retailers stopped selling tobacco products to minors after the full intervention program. Moreover, further 16% retailers never sold tobacco products to minors. Under a fifth (18%) of retailers always sold tobacco products to minors, started or restarted selling tobacco products to minors at some point but note 3% of these were not selling tobacco products to minors by stage two. Overall, 74% of retailers were selling tobacco products to minors at baseline, 40% at stage two and 15% at stage three. These represent significant reductions in selling tobacco products to minors (all stage differences *p* < 0.001). There was no significant difference between retailer types at stage one, stage two and stage three.

## 4. Discussion

Tobacco retailers have a strong influence over whether minors have access to cigarettes and consequently develop smoking habits as adults. Thus, tobacco product retailer employers and employees are equally responsible for ensuring that tobacco products are not sold to minors [17]. In this study, empowerment counseling sessions were applied to train owners and employees of retailers that sold tobacco products to minors in refusing such sales. Before empowerment counseling sessions were applied, the tested retailers that sold tobacco products to minors in the first stage exhibited no significant differences to one another. After the first stage, the empowerment elements of building partnerships, listening, and dialogue were provided and the strategies of building partnership, conducting consensus meetings, and providing a consultation hotline were applied in the counseling process. The reflection, action, and feedback of empowerment elements were provided and the strategies of identifying issues, self-management and completing the reflection and feedback forms were applied in the counseling process. This was the main reason for the significant decrease in the proportion of retailers that sold tobacco products to minors after interventions. The findings of this study revealed that retailers who received empowerment counseling sessions could reduce tobacco sales to minors. The present results are consistent with previous findings [18,19], which concluded that the effects of counseling programs could reduce tobacco sales to minors.

During the present study period, some retailer owners were difficult to approach or ownership changed frequently. The owners of the betel nut stalls that sold tobacco products to minors were so difficult to approach that the first step of the empowerment counseling session, namely building partnerships, was discounted in terms of good performance. The Taiwan Tobacco and Liquor Corporation could construct a database of tobacco product retailers and store owners for training, supervision, and auditing for systematic tobacco hazard prevention. The empowerment counseling session emphasized reflection and feedback to motivate the retail owners to not sell tobacco products to minors. Some betel nut stall owners asserted that adolescents may think it is easy to purchase tobacco products at betel nut stalls because they might adopt lenient approaches to checking the age of tobacco customers, and stated that although not selling tobacco products to adolescents may somewhat influence their profits, they definitely would not sell tobacco products to minors because of concern for adolescent health and the environment. Some betel nut stall owners said that after calculating the potential losses due to incurring penalties for selling tobacco products to minors, which could be hundreds of times the profit of selling such products, they had concluded that selling tobacco products to minors was not worth the risk.

The empowerment counseling session included reflection and feedback, all of which facilitated peer learning among the tobacco product retailers. Feedback in terms of empowerment was applied in the third stage, where the Department of Health Bureau rewarded good performers with an “excellent retailer” certificate to encourage them not to sell tobacco products to minors and to share their positive experiences with other retailers. Some owners or employees of tobacco product retailers stated they may neglect to check buyers’ identifications during busy periods. To solve this problem, the self-management strategies shared by the retailers rated as excellent included cashiers wearing stickers that read “No selling tobacco products to minors: stop, observe, and listen” as an incentive to check buyers’ identifications; offering training courses for tobacco hazard prevention for new employees; and repeating the message of “No selling tobacco products to minors: stop, observe, and listen” during shift changeovers. Of these, cashiers wearing stickers and repeating “No selling tobacco products to minors: stop, observe, and listen” during shift changeovers were deemed the most effective approaches. Some owners of excellent tobacco product retailers stated that they would conduct random disguised checks on their own chain store employees once every four months to check whether they were maintaining the objective of self-management to not sell tobacco products to minors because they thought they should make efforts to support the Tobacco Hazards Prevention Act as a member of society. To accomplish the goal of not selling tobacco products to minors, some retailers established a mechanism in the cash register to forbid sales of tobacco and liquor products to teenagers in 2017. Once the mechanism is activated by the scanning of tobacco or liquor products, an alarm message in red appears on the screen to remind the cashier to verify the buyer’s age. Although this measure may increase a company’s budget, it is highly effective in preventing tobacco products from being sold to minors. Such retailers exhibited a willingness to protect adolescent health and the environment through their positive efforts.

This study provided a basic curriculum for tobacco product retailer owners and employees of various age groups and educational levels to gain a clear understanding of how tobacco hazards influence adolescent health and the environment. The class content covered topics such as the potential dangers of cigarette smoking to adolescents, the consequence of cigarette smoking raising the National Health Insurance budget burden, and the negative influence of tobacco hazards on the environment. Enhancing the efficiency of empowerment strategies counseling reduced the number of retailers that sold tobacco products to minors. Retailer owners and employees were required to be motivated to collaborate with the government and build partnerships—for instance, conducting the educational tobacco hazard prevention program alongside free health-related screening activities such as adult health checks, oral cavity checks, and PM2.5 checks. Tobacco product retailer owners and employees are motivated to attend the educational tobacco hazard prevention program and their willingness to build partnerships is elevated; therefore, they are more likely to accept empowerment counseling sessions. In addition to education on tobacco hazard prevention provided, the health consultation hotline service can facilitate interaction between retailer owners and employees to build partnerships. If the owners of retailers that sold tobacco products to minors cannot be contacted, the employees of those retailers are requested to distribute the Tobacco Hazards Prevention Act flyers and contract to their owners to obtain informed consent and the signed contract. Despite informing the retailers in the study about the Tobacco Hazards Prevention Act, if they continued to violate the agreements in the contract by selling tobacco products to minors, the Department of Health Bureau imposed fines for contract violations.

After the three stages of this study, 19 (6%) of the 327 retailers continued to sell tobacco products to minors, thereby demonstrating that the empowerment counseling program in this study still had room for improvement. Listening and dialogue were the most crucial skills for achieving the objective of the empowerment counseling session [20]. The positive effects of counseling were nullified if the health care providers delivered messages with a tone of excessive power, authority, and command. Some retailer owners and employees reflected that dialogue with registered nurses or counseling volunteers lacked the aspect of listening and made them feel demotivated because of the powerful and commanding tone of the health care providers. Therefore, some owners and employees were hesitant to engage in further interaction with health care providers, therefore, dialogue with mutual communication could not be generated. Consequently, the consensus of “No selling tobacco products to minors” could not be reached and the effectiveness of counseling was nullified. The findings of this study revealed that 6% of tobacco product retailers continued to sell tobacco products to minors and were not influenced by the empowerment counseling program. Thus, it is recommended that health care providers be trained in the skills of listening and dialogue with mutual communication so that they can apply these skills to counseling and assist retailers in identifying their problems and internally building self-awareness and self-management to prevent further sales of tobacco products to minors.

Continual application of empowerment counseling sessions may be one reason for the reduction in the proportion of retailers that sold tobacco products to minors. One of the main features of this study was the reflection and feedback form, which provided tobacco product retailers with the opportunity to not only share their learning experiences, but also participate in opinion exchanges and interactions with other retailers and health care providers. The form also provided a communication channel for tobacco product retailers to ask for help. As this study was limited by insufficient manpower and funds, the empowerment counseling program was applied only to retailers in Southern Taiwan. Future studies could expand the research scope to other counties and cities to offer a more representative image of the situation in Taiwan. Although this study investigated tobacco product retailers in mountainous, coastal, and plain regions, the implemented empowerment counseling sessions may have overlooked differences between rural and urban areas due to different social, economic, and cultural backgrounds, which could influence people’s responses to the counseling sessions. Therefore, empowerment counseling sessions should be designed based on region-centered planning to provide different regions with unique counseling strategies to prevent the selling of tobacco products to adolescents. Finally, experimental design studies could be used to examine the empowerment counseling program for preventing tobacco sales to minors in the future.

To minimize opportunities for adolescents to acquire tobacco products and protect adolescent health, as a government authority, the Department of Health should continue to trace the sources that supply tobacco products to adolescents. For retailers who make such sales frequently, the empowerment strategy of self-management should be enhanced, especially for betel nut stalls. To improve the self-management of retailers not to sell tobacco products to minors and discourage adolescents from using tobacco products and reducing exposure to tobacco smoke, the Ministry of Health and Welfare in Taiwan could develop programs for the plain packaging of tobacco products [21] to reduce tobacco consumption and exposure to secondhand smoke in adolescents. The government could leverage the resources of schools and communities to form voluntary teams for tobacco hazard prevention so that retailers who sell tobacco products to minors within a radius of one kilometer from any junior or senior high school in Taiwan could be consistently monitored and recorded. Through such measures, a safe and carefree living environment can be developed. The power of the Ministry of Education should be utilized to provide adolescent smokers with educational programs about the dangers of smoking and adolescent nonsmokers with preventive education to promote the importance of tobacco hazard prevention. Furthermore, all people can contribute to protecting adolescent health and maintaining a healthy environment with high air quality. 

## 5. Conclusions

Health authorities worldwide play a critical role in encouraging tobacco products retailers to practice self-awareness and self-management to prevent the selling of tobacco products to people aged less than 18 years. In this study, the results revealed the effects of empowerment counseling sessions on reducing tobacco sales to minors. Building partnerships, listening, dialogue, reflection, action, and feedback, as well as the strategies of motivation, encouragement, self-awareness, identifying problems, objective setting, planning, implementation, behavioral changes, reviewing, and maintaining self-management, were all used by retailers that sold tobacco products to minors in Southern Taiwan. In the empowerment counseling program, signing a contract to set an execution objective and collaboration between the government and retailers through reflection and feedback were the most effective in preventing tobacco products from being sold to minors. To improve the empowerment counseling outcomes and reduce the proportion of tobacco product retailers that sell tobacco products to people aged less than 18 years, health care providers should have counseling skills as part of their job education. Moreover, health care providers should collaborate with tobacco product retailers to design unique empowerment counseling sessions for specific regions to improve retailers’ capabilities for self-management in terms of tobacco hazard prevention and promoting a healthy environment for society at large. 

## Figures and Tables

**Table 1:** Empowerment counseling sessions for tobacco retailers that sold tobacco products to people aged less than 18 years.

Empowerment	Empowerment Elements	Counseling Sessions
Motivation, encouragement, and self-awareness: encouraging tobacco product retailers to develop self-management capabilities and self-awareness regarding the importance of not selling tobacco products to people aged less than 18 years.	Building partnerships Listening Dialogue Reflection	Initiate social gatherings to build partnerships. Conduct consensus meetings covering the topics of tobacco hazard prevention (focusing on Article 13 of the Tobacco Hazards Prevention Act), empowerment counseling programs, and encouraging retailers to develop approaches to achieve the goal of not selling tobacco products to people aged less than 18 years. Provide retailers with a booklet entitled “No Selling Tobacco Products to Minors: Stop, Observe, and Listen”, which includes multimedia teaching materials. “Stop” refers to stopping illegal sales of tobacco products to minors. “Observe” refers to discerning whether a customer is a minor and requesting identification if in doubt. “Listen” refers to retailers informing customers of relevant regulations and advising customers to abide by them [16]. Provide a consultation hotline for questions regarding tobacco hazard prevention and empowerment counseling programs.
Inviting retailers to actively participate in identifying concerns to raise their awareness of what are the most crucial concerns that should be resolved first, and providing assistance.	Listening Dialogue Reflection Action: Discern problems to be solved	Registered nurses and counseling volunteers visit retailers: Listen to retailers that sold tobacco products to minors to learn their concerns and the key issues affecting them. Encourage tobacco product retailers to identify problems to be solved. Prioritize problems to be solved by tobacco product retailers. Examples of such problems included newly hired employees lacking professional experience, a lack of clarity regarding the legal status of selling tobacco products to people aged less than 18 years, and not displaying tobacco hazard prevention posters in easily noticeable locations.
Initiating dialogue and discussions with retailers to clarify their problems, and providing them with resources to help them to understand the dangers of selling tobacco products to people aged less than 18 years.	Listening Dialogue Reflection Action: Discern problems to be solved Develop action strategies	Registered nurses and counseling volunteers visit retailers: Listen to retailers that sold tobacco products to minors to learn their concerns and the key issues affecting them. Provide retailers with a tobacco hazard prevention education program, including aspects such as the Tobacco Hazards Prevention Act, tobacco hazards versus adolescent health, and tobacco hazards versus air quality. Invite retailers to sign a contract covering the Tobacco Hazards Prevention Act. The contract includes a penalty for selling tobacco products to people aged less than 18 years. Encourage retailers to design self-management schedules, including objectives and their expected level of effectiveness.
Encouraging tobacco product retailers to develop critical thinking and good decision-making skills through reflection and reviewing their self-management experiences. Working with retailers to identify the problems that should be given top priority and establishing objectives and schedules to solve them.	Reflection Action: Discern problems to be solved Develop action strategies Implement action strategies	Registered nurses and counseling volunteers visit retailers: Ensure that tobacco product retailers are able to develop self-management strategies. Retailers should set rules and corresponding penalties for employees who sell tobacco products to people aged less than 18 years. Adhere to the aforementioned contract in implementing Article 13 of the Tobacco Hazards Prevention Act, with penalties based on sales times. Encourage tobacco product retailers to complete a “reflection and feedback” form related to the empowerment strategies counseling program and tobacco hazards prevention-related topics.
Encouraging retailers to review their self-management experiences and consider the potential effects of behavioral changes to enable their behaviors and responsibilities to be maintained consistently.	Listening Dialogue Reflection Action: Implement action strategies Feedback	Registered nurses and counseling volunteers visit retailers: Continually coach retailers to understand key issues and assist them in developing self-management and self-reflection capabilities to enable them to personally address issues, manage stress, and seek social support. Conduct seminars for achievement sharing and invite all tobacco product retailers in southern Taiwan to participate. The government issues “excellent retailer” certificates to retailers that do not sell tobacco products to minors and encourages such retailers to share their positive experiences with others. In addition, retailers could receive feedback from their peers.

**Table 2:** Retailers sold tobacco products to minors at each stage.

Common	Groups	Whether Retailers Sold Tobacco to Minors at:			N Interventions	N Retailers	% Retailers
	Group Description	Stage 1 Baseline	Stage 2 Post Intervention 1 *	Stage 3 Post Intervention 2 **			
Most common	Stopped selling stage 1	Yes	No	No	1	133	41%
	Stopped selling stage 2	Yes	Yes	No	2	83	25%
	Never sold to minors	No	No	No	0	52	16%
	Always sold to minors	Yes	Yes	Yes	2	19	6%
	Sold to minors stage 2 and 3	No	Yes	Yes	1	18	5%
	Sold to minors stage 2 only	No	Yes	No	1	11	3%
	Sold to minors except stage 2	Yes	No	Yes	1	6	2%
Least common	Sold to minors stage 3 only	No	No	Yes	0	5	2%
						327	100%
						McNamars test (*p*)	
Retailers	N total	% retailers selling tobacco to minors			Stage 1 vs. Stage 2	Stage 2 vs. Stage 3	Stage 1 vs. Stage 3
All retailers types	327	74%	40%	15%	<0.001	<0.001	<0.001
Convenience stores	70	74%	34%	14%	<0.001	<0.001	<0.001
Betel nut stalls	138	78%	41%	24%	<0.001	<0.001	<0.001
Traditional grocery stores	119	68%	42%	13%	<0.001	<0.001	<0.001
*p* (chi^2^ comparing retailers type)		NS	NS	NS			

* Intervention 1 given to retailers selling tobacco to minors at baseline; ** Intervention 2 given to retailers selling tobacco to minors at stage 2; NS not significant (*p* ≥ 0.05).
